# Honor Values as Identity Content: Evidence From a Three-Wave Longitudinal Study

**DOI:** 10.1177/00220221241230959

**Published:** 2024-02-22

**Authors:** Giovanni A. Travaglino, Maria-Therese Friehs, Patrick Ferdinand Kotzur, Dominic Abrams

**Affiliations:** 1 Royal Holloway, University of London, UK; 2 FernUniversität in Hagen, Germany; 3 Durham University, UK; 4 University of Kent, UK

**Keywords:** cultures of honor, honor values, reputation, social identity, regional identification

## Abstract

Reputation refers to the set of judgments a community makes about its members. In cultures of honor, reputation constitutes one of the most pressing concerns of individuals. Reputational concerns are intimately intertwined with people’s social identities. However, research has yet to address the question of how honor-related reputational concerns are structured at the within-person level vis-à-vis individuals’ identification with relevant group memberships. The present longitudinal study investigated the association between social identification and reputational concerns in southern Italy (*N_1st-wave_* = 1,173), a little-studied culture of honor. Specifically, using a random intercept cross-lagged panel model, we tested whether reputational concerns predict, are predicted by, or are bidirectionally linked to individuals’ identification with their region, a group membership relevant for the endorsement of honor. Findings revealed a positive association at the within-person level between group identification and subsequent honor-related concerns. Longitudinal paths from reputational concerns to identification were not significant. Implications of the findings and directions for future research are discussed.

Whether true or false, what is said about [people] often has as much influence on their lives, and particularly on their destinies, as what they do.

—Victor Hugo, “Les Misérables”

Honor has a dual meaning (Pitt-Rivers, 1966). It may denote how people view themselves, that is, the importance they give to adhering to standards of morality. It can also refer to reputation, namely the set of judgments a community makes about its members. As acknowledged by the writer Victor Hugo, reputation is ubiquitously important (Emler, 1990). However, in cultures where honor values are prevalent, so-called cultures of honor, reputation becomes one of the most pressing and crucial concerns of individuals (Rodriguez Mosquera, 2022; Uskul et al., 2019). In cultures of honor, people carefully manage their reputation, even using violence to protect it against threats and insults (Rodriguez Mosquera et al., 2002b).

People’s honor is profoundly intertwined with their social identities. Individuals only have a reputation to the extent that they feel connected to others whose judgment they consider important (Emler, 1990; Levin et al., 2015; Uskul et al., 2019). Moreover, individuals may respond very defensively against acts that question, threaten, or are disrespectful toward the reputation of relevant groups (Barnes et al., 2014). Surprisingly, however, very little research has directly investigated the relationship between honor endorsement and group identification (Barnes et al., 2014; Maitner et al., 2017).

In this study, we leveraged a large longitudinal data set of adolescents in southern Italy to address the question of whether and how individuals’ group identification and honor values affect each other over time. More specifically, we assessed whether individuals’ identification with their region, a group membership relevant to the endorsement of honor in the Italian context (Travaglino et al., 2015), is associated with honor-related concerns for reputation. We employed a random intercept cross-lagged panel model (RI-CLPM) to investigate this relationship at the within-person level of analysis (Hamaker et al., 2015; Mulder & Hamaker, 2021). By focusing on this level of analysis, we were able to draw inferences about how honor values are organized within individuals with respect to their identification with a relevant group. By contrast, methods that focus on between-person variation, or that cannot separate within- from between-person effects (e.g., cross-sectional studies), are unable to shed light on how processes linking values and identities are organized intraindividually (cf. Brandt & Morgan, 2022). Thus, the RI-CLPM is distinctively suited to test whether changes in the endorsement of honor values predict or are predicted by changes in social identification or whether there are bidirectional associations between the two constructs.

## Cultures of Honor

Cultures of honor have developed in many areas around the world, including the southern regions of the United States (Cohen & Nisbett, 1994), South America (Johnson & Lipsett-Rivera, 1998), and Mediterranean countries such as Italy, Spain, and Turkey (Knight & Nisbett, 2007; Rodriguez Mosquera et al., 2002a; Travaglino et al., 2016; Uskul et al., 2012). Historically, cultural codes of honor emerged in environments where the absence of a central authority capable of preventing conflicts required communities to develop their own strategies of social control (Cohen & Nisbett, 1994, J. Schneider, 1971). In such environments, it was essential to affirm one’s own social standing to dissuade potential aggressors. Thus, reputation was an extremely valuable resource that people carefully cultivated and defended (Bowman, 2006).

Although the historical conditions that made honor necessary have typically changed, the endorsement of honor values continues to have important social implications (Uskul et al., 2022). In contexts where honor is prevalent, individuals are more likely to behave in ways that can enhance their reputation while displaying more sensitivity to threats against their social image. For instance, in southern states of the United States, rates of accidental deaths are higher than those in other states (Barnes et al., 2012). This finding has been attributed to a greater propensity to engage in risky behaviors that are perceived to strengthen individuals’ reputation. Indicators of school violence (Brown et al., 2009) and suicide rates (Osterman & Brown, 2011) also tend to be higher in southern states compared with the rest of the United States, presumably indicating a stronger disposition toward aggression and a hypersensitivity to mental health stigma (see also Brown et al., 2014).

In southern Italy, another area where cultural codes of honor are prevalent, individuals’ endorsement of honor values has important implications for their relationships with authorities. In this context, the endorsement of honor-related values of masculinity is associated with stronger support for mafias, violent and criminal groups capable of taking control of territories and exercising authority over communities (Travaglino et al., 2015). This finding is explained by Intracultural Appropriation Theory (ICAT, Travaglino & Abrams, 2019), which posits that groups like mafias can appropriate and make strategic use of cultural values shared in the community to gain legitimacy. Mafia groups go to great lengths to appear as the embodiment of masculinity, power, and honorability, building a reputation as “men of honor” and protectors of the community. By acting in this way, they are able to gain legitimacy among those who endorse honor-related values.

## Honor Values, Groups, and Social Identities

Research indicates that the endorsement of honor values is implicated in people’s relationships with relevant groups. Across cultural contexts, groups constitute an essential feature of human sociality (Brewer & Yuki, 2007). When individuals identify with a group, their sense of self becomes partially defined by their group membership (Hogg & Abrams, 1988; Tajfel & Turner, 1986). Viewing oneself as a group member is associated with incorporating the norms and values of the group into the self-concept (Turner et al., 1987). Thus, threats to the social image of one’s own group may be perceived as threats against the self, triggering similar defensive responses.

Consistent with the idea that there exists a link between honor values and social identity, Barnes et al. (2014) proposed that honor endorsement would foster a “tribe mentality,” which in the American context would lead people to connect with the national group membership. Barnes et al. (2014) found that honor endorsement predicted heightened national identification. National identification, in turn, mediated the relationship between endorsement of honor values and defensive responses against perceived threats against the nation, operationalized as illegal immigration or terrorism. Maitner et al. (2017) reported that individuals from an honor culture (United Arab Emirates) were more likely to display heightened emotional reactions to insults against their ethnic identity, which in the Arab context is an honor-relevant identity, than individuals from a nonhonor culture (Great Britain). By contrast, Arab and British participants both had similar reactions to insults against their identity as students, which is less honor-relevant in the Arab context. Rodriguez Mosquera (2018) also found a correlation between individuals’ identification as British Muslims and honor values.

Finally, Travaglino and colleagues investigated the link between regional identification and honor values in Italy (Travaglino et al., 2015, 2017). Italy is a relatively younger nation state, which officially unified in 1861. Regionalism has an especially pronounced importance in the Italian context, shaping identities, languages, and political relationships (Cavazza, 2012; Levy, 1996). Anthropological work has shown the relevance of cultural codes of honor for regional identities in the south of Italy (P. T. Schneider, 1969). Consistent with this work, Travaglino and colleagues reported an association between southern Italians’ identification with their region and their endorsement of honor values (Travaglino et al., 2015, 2017). Honor, in turn, mediated the association between the strength of their regional identification and their support for mafia groups, which portray themselves as protectors of the local community.

Overall, findings from these studies support the idea that individuals’ social identity and their endorsement of honor values are associated across cultures, groups, and contexts. However, these studies have yet to investigate the very crucial question of how honor values and social identities are interconnected and organized within individuals. Specifically, research has yet to assess whether changes in people’s endorsement of honor values instigate changes in their identification with a relevant group (as hypothesized in Barnes et al., 2014) or, alternatively, because adopting honor values may provide meaning to a specific group membership, it is changes in identification that invoke changes in the adoption of honor values (a perspective more compatible with the work of Maitner et al., 2017 and Travaglino et al., 2017). We examined these alternative perspectives in the current research.

These two perspectives map onto different sociocognitive pathways that may link values to group identification (Van Veelen et al., 2016). The perspective conceptualizing value as an antecedent to social identification presupposes a process of self-anchoring, according to which elements that are central to the self are projected onto a relevant group. In turn, self-anchoring contributes to creating a bond between individuals and the group, strengthening their social identification (Cho & Knowles, 2013; Otten & Epstude, 2006, cf. also Wan et al., 2007). Conversely, viewing identification as an antecedent of value endorsement assumes a process of self-stereotyping through which characteristics of the group are assimilated into the self. The self-stereotyping pathway implies that when the group becomes more relevant to the self, individuals tend to incorporate group characteristics into the self-concept (Hogg & Abrams, 1988; Hogg & Turner, 1987; Turner et al., 1987). Thus, identification with the group may strengthen the endorsement of values that are relevant to the group.

In principle, both sociocognitive pathways are compatible with social identity theory (Van Veelen et al., 2016). They may each operate under specific circumstances (e.g., Van Veelen et al., 2013) or in parallel, involving mechanisms of feedback loop whereby value endorsement and identification are mutually reinforcing. So far, very little research has explicitly compared these two pathways in the context of individuals’ endorsement of cultural values (e.g., Wan et al., 2007), and none in the context of honor-related values.

Previous studies were unable to tackle the question of how honor is organized with respect to identity because the cross-sectional methods employed—correlations and experiments—cannot disentangle between-person differences from within-person effects (Brandt & Morgan, 2022; Fisher et al., 2018; Hamaker, 2012). That is, it remains unclear whether the cross-sectional relationships observed indicate the existence of differences *between* people (e.g., people who identify more strongly with their group than others also tend to place greater value on their honor than others) or instead speak to the existence of intraindividual processes (i.e., someone who identifies more strongly with their group will subsequently place greater value on their honor). Notably, inferences obtained at one level of analysis only very rarely generalize to the other (Hamaker, 2012). In this research, we employed an analytical technique that can distinguish between-person variance from within-person variance in longitudinal data, the RI-CLPM (Hamaker et al., 2015). By employing the RI-CLPM, we were able to address for the first time the question of how fluctuations around individuals’ trait levels of either identification or honor endorsement are prospectively linked to changes in either of the two constructs (see also Orth et al., 2021). Specifically, we were able to address the question of whether individuals’ social identification precedes, is preceded by, or is mutually linked with individuals’ endorsement of honor values.

Investigating the ways honor values and identity are organized within individuals is crucial because it can also help clarify how honor endorsement is shaped by group dynamics. For instance, if honor is the content of a specific social identity, the way in which honor will influence behavior will depend on the wider set of intergroup relations (Hogg & Abrams, 1988). Specifically, honor may lead to different behaviors depending on which group is seen as “us” or “them.” This could explain why honor has been linked to the defense of the nation in the U.S. context, whereas in southern Italy, it is associated with support for criminal and anti-institutional groups such as the mafia.

## Method

### Participants

Participants were 1,173 (*M_age_* = 16.70, *SD*_age_ = 1.10, 43.8% female, 54.8% male, 1.4% did not specify their gender) residents in Campania, a southern region in Italy. They were recruited from three schools across a single area and took part in a larger study assessing cultural values of honor, identity, and attitudes toward criminal groups (Travaglino et al., 2023). The majority (94.2%) of the participants were born in Campania, 2.4% in other regions and 3.4% did not indicate the region where they were born. The study was ethically approved by the first author’s academic institution at the time of data collection, the University of Kent.

### Procedures and Measures

Data were collected in October 2014 (*n*_Wave1_ = 1,173), February 2015 (*n*_Wave2_ = 1,087), and June 2015 (*n*_Wave3_ = 1,087), during school hours. Two researchers visited classrooms and administered pencil-and-paper questionnaires to pupils. Participants took part in the study voluntarily and completed the measures at their own pace. Cross-sectional analyses employing data from Wave 1 were reported in Travaglino et al. (2017). Longitudinal analyses are reported for the first time in this article. To link questionnaires across waves, participants were asked to generate a personal code by answering some simple questions (e.g., the first letter of the Mother’s surname, the last two digits of their phone number). Participants were debriefed in group discussions at the end of the research project.

Questionnaires were in Italian, and items were measured on a 7-point Likert-type scale (1 = *completely disagree*, 7 = *completely agree*). Four items were used to measure participants’ social identification with the regional group membership (Travaglino et al., 2015), “I am pleased to think of myself as Campano,” “I am proud I am Campano,” “I Identify with other people who live in Campania,” and “I feel a sense of belonging to Campania.” Five items were used to measure participants’ endorsement of honor values (Rodriguez Mosquera et al., 2008). The items were, “it is important to me that others see me as someone who deserves respect,” “it is important to me that others regard me as someone who is not to be disrespected,” “how others think of my family is important to me,” “caring about the implications of my actions for my family’s social image is important to me,” and “it is important to me to defend my family from criticism.”^1^ Table 1 reports the reliability estimates (ω) for the measures across waves.

**Table 1.** Descriptive Statistics and Intercorrelations for the Mean Values of All Constructs Across All Time-Points.

	*N*	*M*	*SD*	Minimum	Maximum	1	2	3	4	5	6
Honor Wave 1	1,172	5.26	0.94	1.00	7.00	.695					
Honor Wave 2	1,087	5.16	0.98	1.00	7.00	.632	.777				
Honor Wave 3	1,086	5.04	1.01	1.00	7.00	.555	.678	.798			
Identity Wave 1	1,172	4.85	1.19	1.00	7.00	.216	.238	.179	.822		
Identity Wave 2	1,087	4.79	1.18	1.00	7.00	.181	.281	.211	.689	.841	
Identity Wave 3	1,087	4.72	1.19	1.00	7.00	.191	.244	.239	.653	.725	.869

*Note*. The diagonal line includes reliability estimates (omega/ ω), while the intercorrelations are depicted below the diagonal. Scale ranges from 1 = *strongly disagree/not at all likely* to 7 = *strongly agree/extremely likely*. Honor = honor values, Identity = regional identity. All the correlations are significant at *p* < .001.

## Results

### Preliminary Analyses

We first checked whether systematic attrition across waves occurred across demographic variables, social identification, and honor value items utilizing a series of analysis of variance (ANOVA) and Chi-square test with a Bonferroni-corrected *p* < .0042 (*p* = .05/12 comparison per set of tests).^2^ Only gender emerged as significant in these series of tests for attrition from Waves 1 and 2 (*p* = .003) and Waves 2 to 3 (*p* < .001), indicating the need to include gender in the model to reduce potential systematic bias in estimated values. In addition, we ran a series of Little’s missing completely at random (MCAR) tests for each wave. None of these tests were significant (Wave 1: χ^2^ = 61.725, *df* = 77, *p* = .898, Wave 2: χ^2^ = 16.127, *df* = 43, *p* = 1.000, Wave 3: χ^2^ = 58.173, *df* = 68, *p* = .796), indicating that within-wave missingness did not follow any observable pattern and could be assumed to be completely at random. To reduce model complexity in the RI-CLPM, we used composite scores derived from averaging the scale indicators. The comparison of composite scores across waves requires us to satisfy the assumption of scalar measurement invariance (i.e., factor loadings and intercepts are equal across waves, Little, 2013). Using a stepwise process and employing Chen’s (2007) ΔCFI = .01 criterion, we were able to successfully establish the existence of equal loadings, Δ $CFI_{Honor}$ = .002 and Δ $CFI_{Identification}$ < .001, and equal intercepts Δ $CFI_{Honor}$ = .005 and Δ $CFI_{Identification}$ = .001, for both measures.

### Testing the RI-CLPM

We first modeled a cross-lagged panel model (CLPM). The CLPM formed the basis of the RI-CLPM, and we used it to establish stationarity assumptions (i.e., “the degree to which one set of variables produces change on another set remains the same over time,” Cole & Maxwell, 2003, p. 560). Analyses were conducted using r software (R Core Team, 2022), and the packages “lavaan” (Rosseel, 2012), “semTools” (Jorgensen et al., 2022), “psych” (Revelle, 2023), tidyverse (Wickham et al., 2019), and MBESS (Kelley, 2022). Using a stepwise process (Swart et al., 2011), we first established equality constraints of the stability (autoregressive) coefficients of each construct. Subsequently, we imposed equality constraints for the cross-lagged effects between the different constructs over time. These increasingly restrictive models were examined using the Satorra–Bentler scaled χ^2^ difference test (Satorra & Bentler, 2001). A nonsignificant result implies that model restrictions can be applied without substantially impairing model fit. Stationarity assumptions were supported for stability coefficients, $\Delta \chi^{2}$ (2) = 4.391, *p* = .111, and cross-lagged effects, $\Delta \chi^{2}$ (2) = 4.727, *p* = .094.

The CLPM was subsequently transformed into a RI-CLPM, following the instructions of Mulder and Hamaker (2021). The RI-CLPM separates the observed variance of the indicators into a random intercept and latent wave factors. The random intercept represents stable between-person differences, whereas the latent wave factors indicate within-person fluctuations around a participant’s trait level of the construct. The latent wave factors are then used to model autoregressive and cross-lagged relationships, which can be interpreted as pure within-person effects. Latent wave factors are modeled without association with the random intercepts, while the random intercepts of the constructs are allowed to covary. Participants’ gender and age were added as predictors of the manifest indicators to control for the effects of these variables.

The model fits the data well, root mean square error of approximation (RMSEA) = .037, standardized root mean square residual (SRMR) = .016, and comparative fit index (CFI) = .996 (Schermelleh-Engel et al., 2003). Model coefficients are summarized in Table 2. Note that autoregressive and cross-lagged paths are stationary, yielding the same unstandardized coefficients, confidence intervals, and *p* values across waves (cf. Usami, 2021). Slight variations in the standardized coefficients are due to changes in the total amount of variability across waves employed to standardize them.

**Table 2.** Random-Intercept Cross-Lagged Panel Model Coefficients.

Path	β	*b*	*SE*	*p* value	95% CI
Between-person effects					
Identification ←→ Honor	.260	.164	0.036	<.001	
Within-person effects (Wave 1 → Wave 2)					
Identification → Honor	.126	.123	0.052	.018	[0.021, 0.225]
Honor → Honor	.269	.292	0.071	<.001	[0.154, 0.430]
Identification → Identification	.199	.204	0.074	.006	[0.059, 0.349]
Honor → Identification	.098	.111	0.059	.062	[−0.006, 0.227]
Within-person effects (Wave 2 → Wave 3)					
Identification → Honor	.126	.123	0.052	.018	[0.021, 0.225]
Honor → Honor	.286	.292	0.071	<.001	[0.154, 0.430]
Identification → Identification	.211	.204	0.074	.006	[0.059, 0.349]
Honor → Identification	.110	.111	0.059	.062	[−0.006, 0.227]
Time-invariant predictors					
Gender → Honor (W1)	.129	.251	0.055	<.001	[0.144, 0.358]
Age →Honor (W1)	−.080	−.068	0.024	.005	[−0.116, −0.020]
Gender → Honor (W2)	.115	.232	0.058	<.001	[0.120, 0.345]
Age → Honor (W2)	−.125	−.111	0.026	<.001	[−0.162, −0.059]
Gender → Honor (W3)	.068	.139	0.059	.018	[0.024, 0.254]
Age → Honor (W3)	−.113	−.100	0.026	<.001	[−0.151, −0.049]
Gender → Identification (W1)	−.049	−.119	0.067	.074	[−0.251, 0.012]
Age → Identification (W1)	−.202	−.213	0.030	<.001	[−0.272, −0.155]
Gender → Identification (W2)	−.063	−.152	0.068	.026	[−0.285, −0.018]
Age → Identification (W2)	−.159	−.168	0.031	<.001	[−0.228, −0.107]
Gender → Identification (W3)	−.064	−.153	0.068	.024	[−0.286, −0.020]
Age → Identification (W3)	−.153	−.159	0.030	<.001	[−0.218, −0.101]

*Note*. Honor = honor values, double-headed arrows (←→) are covariances. One-headed arrows (→) are regression coefficients. Unstandardized stability and cross-lagged paths were stationary across waves, small differences in the beta coefficients depend on differences in the total variability of the criteria variables which can change across waves. Gender was coded 0 = *male*, 1 = *female*. Age was centered to its mean. CI = confidence interval.

The standardized factor loadings of honor were slightly higher for the between-person factors (ranging from .76 to .78) compared with within-person factors (ranging from .61 to .62), indicating slightly more explained variance for between-person factors. Concerning identification, the between-person factor loading (.71) indicated slightly more explained variance than the within-person factor loading (.68) at Wave 1. At Waves 2 and 3, within-person factor loadings (.71 and .72) indicated slightly more explained variance than between-person factor loadings (.68 and .68).

**Figure 1.**
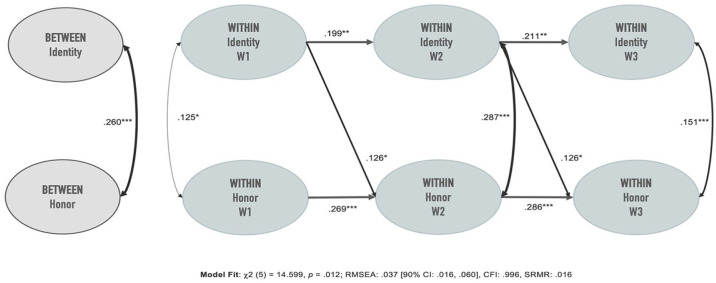
Significant Paths (Standardized) of the Random Intercept Cross-Lagged Panel Model. *Note*. RMSEA = root mean square error of approximation; CI = confidence interval; CFI = comparative fit index; SRMR = standardized root mean square residual.

Figure 1 summarizes the model’s significant paths. At the between-person level, and in line with prior research (e.g., Barnes et al., 2014), there was a significant positive correlation between honor endorsement and identification. Participants who identified more strongly with their regional group membership than others also endorsed honor values more strongly than others. At the within-level, the results were more compatible with the idea that honor constitutes identity content. The cross-lagged path from honor to identity was not significant, while the path from identity to honor was significant. To further test the idea that honor constitutes the content of the regional group membership, we assessed models in which the cross-lagged paths for either of the two constructs were constrained to zero and tested whether doing so significantly deteriorated the fit of the model. Constraining the cross-lagged paths from endorsement of honor values to identification to zero did not significantly deteriorate the fit of the model, $\Delta \chi^{2}$ (1) = 3.426, *p* = .064. Conversely, constraining the cross-lagged paths from identity to honor resulted in a significantly worse model fit, $\Delta \chi^{2}$ (2) = 5.459, *p* = .019.

## Discussion

In cultures of honor, concerns about reputation have very substantial implications for many aspects of people’s social life, including health (Brown et al., 2014), aggression (Brown et al., 2009; Osterman & Brown, 2011), and perceptions of authorities (Travaglino & Abrams, 2019). Prior research has demonstrated the existence of an association between honor and social identification across contexts and groups (e.g., Barnes et al., 2014; Travaglino et al., 2015). This finding is consistent with the idea that honor is deeply intertwined with people’s sense of belonging to relevant groups. In this article, we extended previous research by investigating longitudinally the relationship between individuals’ honor-related concerns about reputation and social identification with a relevant regional group membership. Specifically, we examined whether and how individuals’ honor and identification affect each other over time. By employing an RI-CLPM to analyze our data, we were able to disentangle the between- from within-person level of analysis and address the critical issue of how beliefs about identity and honor are organized within individuals.

We tested different perspectives derived from previous research. Previous studies have conceptualized honor either as fostering identification with relevant groups (Barnes et al., 2014) or as the content of specific social identities (Maitner et al., 2017; Travaglino et al., 2015). Notably, these perspectives map onto different sociocognitive pathways linking identification to values, namely self-anchoring and self-stereotyping, respectively (Van Veelen et al., 2016). As these pathways are not mutually exclusive, it is also possible that individuals’ identification with relevant groups and their endorsement of key values are mutually constitutive and bidirectionally linked.

Overall, results from our three-wave longitudinal study were more compatible with the notion of honor as social identity content, providing evidence for the self-stereotyping pathway. Before being separated into the within- and between-person variances, we observed associations between participants’ age, gender, and their endorsement of honor values and identification with the regional group membership. Younger male participants were more likely to endorse honor-related reputation concerns than older female participants. This finding is consistent with prior research highlighting the importance of reputation during adolescence and for men (Emler, 1990). Moreover, there was also a tendency for younger and male participants to identify more strongly with the regional group, highlighting the importance of local belonging for these categories (cf. Félonneau et al., 2013). The effects of age were typically stronger than the effects of gender (see Table 2).

More central to the objective of this research, at the between-person level, and replicating previous research (Barnes et al., 2014; Maitner et al., 2017; Rodriguez Mosquera, 2018; Travaglino et al., 2015), there was a positive association between identification and honor-related concerns for reputation. At the within-person level, findings revealed significant medium-to-large (Orth et al., 2022) cross-lagged associations from identification to honor across all time intervals. Conversely, cross-lagged associations from honor to identification were not significant. Furthermore, whereas constraining the cross-lagged relationships from identification to honor to zero significantly deteriorated the fit of the model, constraining the cross-lagged relationships from honor to identification to zero did not.

The findings indicate that individuals who reported stronger-than-usual identification with their regional group membership subsequently experienced stronger concerns for reputation (cf. Orth et al., 2021 for a discussion on the interpretation of findings from the RI-CLPM). Notably, the items used in this study tapped into individuals’ concerns for their and their family’s reputation rather than the reputation of the regional group membership (Levin et al., 2015). Thus, the relationship between honor values and identification cannot be merely attributed to the enhanced importance of the group’s image for the self. Instead, this pattern of results suggests that honor values are ascribed to the self in response to changes in the saliency of a relevant group membership, consistent with a self-stereotyping mechanism. In other words, honor can be conceptualized as part of the broader repertoire of norms, values, and ideological frameworks that define the meaning of a specific identity (i.e., what it means for individuals to belong to a group).

The finding that, at within-person level, cultural values are contingent on a given identity offers crucial insights into how the constructs of identity and culture may be organized psychologically (Hopkins & Reicher, 2011). Specifically, the results have important implications for our understanding of honor in the context of both interpersonal processes and (inter)group dynamics. They suggest that contextual factors that may alter the salience of a social identity may also shape the centrality of honor to individuals’ lives, in turn affecting behavior. For instance, the emergence of an honor mindset has been implicated in aggressive and emotional responses against insults (Maitner et al., 2017; Rodriguez Mosquera, 2018; Uskul et al., 2019), the defense of groups from external threats (Barnes et al., 2014), and legitimizing attitudes toward criminal authorities (Travaglino et al., 2015). Such an honor mindset may be predicated on a given identity becoming salient, shaping what individuals perceive as the in-group (Turner et al., 1987).

The RI-CLPM enabled us to address for the first time the question of how individuals’ social identification and endorsement of honor values are organized within individuals. Nonetheless, the study was affected by some limitations. Similarly to other nonexperimental methods, the RI-CLPM does not constitute a complete test of causality. Future research should manipulate the salience or centrality of relevant identities to further investigate its causal impact on the endorsement of honor values, together with honor’s implications for social behavior, attitudes, and other psychological characteristics. For instance, research has shown that individuals’ endorsement of honor is linked to increased well-being when it aligns with the honor endorsement by others in one’s (distal or proximal) social environments (Kirchner-Häusler et al., 2024). Findings from this study suggest that, because honor may constitute the content of relevant social identities, it may unlock the potential of identities to enhance well-being by providing individuals with guidance on the norms and behaviors suitable for their respective contexts (Haslam et al., 2018; Travaglino et al., 2020). Additional research is warranted to understand further the complex interplay between social identity, honor, and well-being.

The present research focused on the relationship between identification and honor-related concerns for reputation. Importantly, in cultures of honor, the notion of reputation is gendered and encompasses different norms and expectations for men and women (Rodriguez Mosquera, 2022). Additional research is required to address the linkages between identification with relevant groups and these other, more specific, facets of honor values.

The generalizability of our findings on the relationships between identification and honor should be tested through research with populations from different geographical areas, with different group memberships, and with different cultures. For instance, it would be crucial to examine how honor is organized with respect to ethnic (Rodriguez Mosquera, 2018) or national (Barnes et al., 2014) identities. Furthermore, research should investigate which contextual factors are responsible for emphasizing honor values and strengthening the identity–culture link, as well as identifying factors that may weaken such a link.

Finally, longitudinal studies conducted over a longer period and across different age groups could help clarify whether the relationship between identification and honor remains consistent over time or changes in response to life events and socialization stages. It should be noted that participants in this study were adolescents, a period especially critical for the formation of identities (McAdams & Pals, 2006), the reinforcement of a sense of affiliation to groups (Tarrant et al., 2001), and the internalization of frameworks of norms and values (Frey et al., 2017; Somech & Elizur, 2009). As children enter adolescence, they become particularly sensitive to threats to their group membership (belonging, Abrams et al., 2017) and increasingly emphasize social conventional (normative) factors in securing their peer relationships, reflecting not only on in-group norms but also on questions of loyalty and the in-group’s differentiation from relevant out-groups (Abrams et al., 2011).

Thus, one possibility is that as people’s sense of self becomes more complex and they are better able to integrate different memberships in their self-concept, they may start identifying more strongly with groups that align with their value preferences instead of endorsing specific values based on the strength of their identification with particular groups (Wan et al., 2007). In addition, the two processes of self-stereotyping and self-anchoring could start operating in parallel, which implies bidirectional relationships between social identification and values (cf. Van Veelen et al., 2016). Indeed, recent evidence suggests that the relative importance of the peer group in determining individuals’ attitudes in other domains weakens but does not disappear as individuals approach adulthood (Seddig, 2020). Thus, although the cross-lagged paths in our model were stationary (implying stability of the process in the temporal arc considered), more studies that disentangle between-person differences from within-person change are needed to address the complex dynamics linking social identity to culture.

## Conclusion

In many cultures, honor values play an essential role in regulating interpersonal and intergroup behavior. These values are tied to social identities and can influence how individuals perceive and respond to threats to their reputation or social standing. Our finding indicates that individuals experience more concerns about reputation when a social identity becomes salient. Honor can, therefore, be conceptualized as the content of relevant social identities. Further research is needed to fully understand the dynamics of the relationship between identification and honor and how it affects individual and group behavior. By examining these issues in greater depth, we can gain a better understanding of how honor values shape social interactions.

## Notes

1. Data set and analysis code are available at the following link: https://osf.io/5uejd/?view_only=57864a0c37884cb28ce6515845585a42

2. The comparisons involved age, gender, region, the four indicators of social identity, and the five indicators of honor values across the three waves (Waves 1–2, and Wave 2–3). Comparisons involved participants who (a) dropped out after participating in a previous wave (e.g., participated in Wave 1, but not in Wave 2), (b) joined at a later wave after not participating in a previous wave (e.g., did not participate in Wave 1, but participated in Wave 2), and (c and d) continued (non)participation across waves (e.g., participated in Waves 1 and 2, and did not participate in Waves 1 and 2).
